# Rural–Urban Differences in Physical Fitness and Overweight Prevalence of Children and Adolescents from Central South China

**DOI:** 10.3390/ijerph20032390

**Published:** 2023-01-29

**Authors:** Qing Jiang, Xin Huang, Di Cui

**Affiliations:** 1 School of Physical Education, Hunan University, Changsha 410082, China; 2 Hunan Province Students’ Physical Fitness Test Data Management Center, Changsha 410082, China

**Keywords:** physical fitness, overweight, children and adolescents, rural-urban, latitudinal

## Abstract

Purpose: This present study aims to determine the rural–urban differences in physical fitness and overweight prevalence among children and adolescents from Central South China. Methods: All the original parameters of physical fitness indicators for 216,718 participants aged from 7 to 15 years old were obtained from the website of the Chinese National Student Physical Fitness Database and were analyzed by SPSS. Results: (1) Children and adolescents from rural areas were observed to have a more adverse physical fitness ratio, and the urban–rural differences were identified in each physical fitness indicator. (2) Rural areas had a higher overweight prevalence (*p* < 0.01). When compared to urban cities, overweight students from rural towns measured a significantly poorer cardiorespiratory and muscular fitness in primary school (*p* < 0.05), while the result in middle school was showed the opposite. (3) Rural–urban residence and sex were the moderately correlated factors for muscular fitness among overweight students. Conclusions: Urban children and adolescents in central south China had an overall healthier profile than their rural peers, particularly in overweight groups. The government and related functional departments should take the factors of rural–urban residence and sex of students into consideration when building a state strategy and interventions to promote physical activity and health.

## 1. Introduction

Children’s and adolescents’ physical fitness are important embranchments of public health, and they serving as the adulthood health foundation for individual development and maturation. The governments of countries around the world have adopted different strategies to monitor and improve children’s and adolescents’ physical fitness. In China, the National Student Physical Health Standard (revised 2014, short for standard below) has been launched by the Ministry of Education since 2002 [1], and by collecting and monitoring the physical fitness information of children and adolescents at school age, it aims to advocate the national guiding ideology of health first, strengthen the school physical education work, and promote students to actively participate in physical exercise by developing good exercise habits and improving their levels of physical fitness.

Physical fitness, which is the ability to perform motions or activities, has been widely recognized as a powerful marker of health in children and adolescents [2], and it is positively associated with present and future health-related outcomes. Previous studies showed that a low level of physical fitness usually symbolized a high risk of cardiovascular disease [3], cancer [4], hypertension [5], mental disorder [6], etc. Physical fitness is mainly inherited and genetically determined, but it can also be greatly influenced by geographic and environmental factors [7]. There are studies around the world focusing on the association between place of residence (urban or rural) and physical fitness in children and adolescents, and results show, according to the discrepancy among different countries and regions, that the urban–rural differences in school age students’ physical fitness varies [8,9,10,11,12].

The newly reported data from the World Health Organization (WHO) showed that worldwide obesity had nearly tripled since 1975, and over 340 million children and adolescents aged 5–19 were overweight or obese [13]. The annual report on Chinese children’s development (2021) showed that the problem of overweight/obesity rates became increasingly serious from 2010 to 2019, and the overweight/obesity rate of urban students has always been higher than that of rural students [14]. Overweight/obesity rates during childhood are associated with cardiovascular dysfunction and asthma, in addition to psychological problems, including lower self-esteem, poorer achievement, and lower quality of life [15,16,17]. Therefore, physical health monitoring and intervention for overweight/obese children and adolescents should be considered as a public health priority.

2020 is the year of decisive victory in building a moderately prosperous society in all respects and fighting against poverty in China. It is of great value to look deep into the urban–rural differences in physical fitness in children and adolescents for future health promotion-related policy formulations. Besides, the global pandemic of Coronavirus disease 2019 (COVID-19) had a dramatic impact on people’s life behaviors due to movements restrictions, and, in China, particularly in central south China, the outbreak led to an unprecedented home quarantine for at least 6 months among students in 2020. The curriculum of physical education (P.E.) was taught online, and the impact of regional epidemic responses on student physical activity and physical fitness was different [18]. Based on the above contexts, this present study explored the characteristics of the physical fitness indicators of children and adolescents in rural and urban areas from central south China and examined the rural–urban differences in physical fitness and overweight prevalence of children and adolescents after the COVID-19 epidemic, with the aim to provide a reference for building a precise state strategy and interventions to promote physical activity and health level in school aged youth.

## 2. Methods

### 2.1. Source of Data

The data were authorized and obtained from the Chinese National Student Physical Fitness Database, and the original parameters of physical fitness indicators were used to assess children’s and adolescents’ fitness. The present study analyzed the physical fitness data of 216,718 students (112,923 boys, and 103,795 girls), aged from 7 to 15 years old, who were respectively distributed among different rural and urban areas of Changsha, which is a represented city of central south China. All the data recruited in the present article were collected during the winter semester in 2020–2021 at school during school hours. According to the adolescent growth and development characteristics and the Chinese education system, the student physical fitness data were analyzed according to school grading system in elementary school (grade 1st–2nd, 3rd–4th, and 5th–6th) and middle school (grade 7th–9th). The ratio of residences from urban and rural areas was close to 1:1 (Table 1).

### 2.2. Study Design

This present study was an original article, all the data were approved by the Chinese Ministry of Education, and formal consent was obtained from the parents and students. The students’ physical fitness data were collected from 1072 primary and middle schools and were analyzed by school grading system in grade 1st–2nd (aged from 7 to 8 years old), 3rd–4th (aged from 9 to10 years old), 5th–6th (aged from 11 to 12 years old), and grade 7th–9th (aged from 13 to 15 years old).

The physical fitness was assessed by parameters indicating students body composition (BMI), speed agility (50 m sprint), cardiorespiratory fitness (vital capacity, 1 min rope skip, middle distance race, or shuttle run), muscular fitness (1 min rope skip or long jump, pull up, or 1min sit up), and flexibility (sit reach). Concretely, according to the Standard, except for the body composition, vital capacity, sit reach, and sprint (50 m), the tested criteria varied among different grades with the 1 min rope skip in all grades of elementary school, the 1 min sit up in grades 3rd–4th and 5th–6th, the shuttle run (50 m × 8) in grade 5th–6th, and the long jump, middle distance race (male 1000 m and female 800 m), and muscle strength (male pull up and female 1 min sit up) in middle school. The overall physical fitness assessment scores were calculated by the formula defined in the Standards by weight of different indicators (Table 2).

Additionally, the overall cardiorespiratory fitness was assessed by the comprehensive analysis of respectively observed indicators according to the Standards, with vital capacity (15%) plus middle distance race (middle school, 20%) or 1 min rope skip (elementary school, 20%), and for grade 5th–6th, an extra 15% of the shuttle run was particularly supplemented. The overall assessment of muscular fitness was the comprehensive of indicators mentioned in Table 2, which included 1 min rope skip in grade 1st–2nd, 20% of 1 min rope skip combined with 10% of 1 min sit up in grade 3rd–4th, 10% of 1 min rope skip combined with 20% of 1 min sit up in grade 5th–6th, and 10% of male pull up/female 1 min sit up combined with 20% middle distance race and an extra 10% of long jump in grade 7th–9th. All the measurements were carried out at school during school hours, and test scoring and overall fitness performance were assessed and judged with scores of excellent (scores 90 or above), good (scores 80–89.99), pass (scores 60–79.99), and fail (scores below 60). The value of BMI was classified into low weight, normal weight, overweight, and obesity according to the Standards, and the sex-and-grade specific BMI cut points of different weight statuses are shown in Table 3.

**Figure 1:**
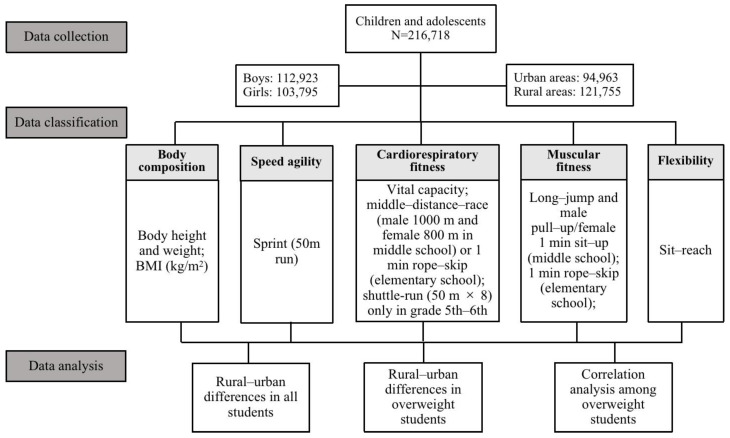
Study flowchart of data collection, classification, and analysis.

The definition and meaning of rural and urban residence vary across geographical areas and countries depending on different national standards [19]. In this present study, the classification of urban and rural residence was based on the revised criteria for designed towns issued in 1984, and it has not been changed since the initial classification in 1985 [20]. Figure 1 shows the overall study flow of the present study (Figure 1).

### 2.3. Statistical Analysis

Means, standard deviations, frequencies, and percentages were calculated to describe body composition and physical fitness indicators for urban and rural groups, for boy and girl groups, and for the total group. The chi-square test was used to measure the rural–urban differences of the ratios (only for excellent or not and for fail or not) of overall physical fitness and of each individual parameter assessment. Two-way ANOVA and multiple comparison were performed to detect differences in urban–rural residence on the original values of the physical fitness parameters. The co-relationships between BMI, sex, urban–rural residence, and each physical fitness parameter were assessed by the analysis of bivariate correlations. Statistical significance was set at *p* < 0.05 or *p* < 0.01, and all analyses were performed using IBM SPSS statistics version 17.0. All the analyzed chi-square values (χ^2^) and F values are shown in the Supplementary Materials.

## 3. Results

### 3.1. Urban–Rural Disparity in Physical Fitness in Overall Samples

Overall Fitness: Urban students achieved 14.28% in “excellent”, 30.35% in “good”, 49.29% in “pass”, and 6.08% in “fail” marks for overall physical fitness performance, and rural students were accordingly 3.34%, 23.78%, 65.71% and 7.17%, respectively (Table 4). The percentage of rural student overall fitness scores of “excellent” was significantly lower (*p* < 0.01, χ^2^ = 8359.961) than their urban peers. The similar result remained in all grades, especially in grades of elementary school (*p* < 0.01, Table 4). Conversely, rural students had a slightly higher “fail” ratio (*p* < 0.01, χ^2^ = 103.513) than urban ones (7.17% versus 6.08%), while in grade 7th–9th, that ratio in urban students (12.31%) was much higher than in rural students (8.72%), which resulted a significantly higher ranked “fail” proportion in rural than that in urban students among all grades of primary school-aged students (*p* < 0.01, Table 3).

Body Composition: The proportions of “low weight”, “overweight”, and “obese” between the rural and urban students were analyzed by the chi-square test, and the results are shown in Table 5. When compared to their urban peers, except for the girls in grade 1st–2nd, students from rural areas had a significantly higher “low weight” proportion and an even lower overweight percentage (*p* < 0.05). Except for the girls in grade 1st–2nd, all the students from urban areas had remarkably wider distributions of “overweight” than rural students (*p* < 0.05). Furthermore, for the distribution of “obesity”, primary school girls from rural areas were detected with markedly higher percentages (*p* < 0.01) than urban ones, while on the contrary, the situation in the middle school was significantly reversed (*p* < 0.05, Table 5).

Speed Agility: The 50 m sprint is a universal physical fitness test to assess one’s ability of speed agility in all grades, and, according to the data presented in Table 6, rural primary school students measured higher ratios for failing this test when compared to urban ones (*p* < 0.05), with the results being reversed in middle school students (*p* < 0.01). Additionally, there were much more rural girls who performed “excellent” during the sprint test than their urban peers, except for grade 1st–2nd (*p* < 0.01), while the situation was reversed in boys (*p* < 0.01).

Cardiorespiratory Fitness: Descriptive data and the rural–urban differences on cardiorespiratory fitness are presented in Table 7. All the students from rural towns measured an even lower “excellent” score and a relatively higher ranked “fail” proportion among all observed grades (*p* < 0.01) in the vital capacity test. The results of the 1 min rope skip resulted showed similarly for the vital capacity, with a much larger gap between rural and urban areas (*p* < 0.01). Additionally, the shuttle run was only measured in grade 5th–6th and, except for a slightly large amount of rural girls who performed in the “excellent” range, all the students form rural towns were ranked much lower in “excellent” and more in “fail” proportions than their urban peers (*p* < 0.05). On the contrary, when testing the middle distance race, there were more students from rural areas who performed in the “excellent” range than urban students (*p* < 0.01), while a much high ratio of urban students failed in this test than rural peers (*p* < 0.01) except for girls. In total, the chi-square test showed that the overall cardiorespiratory fitness in rural areas was significantly poorer than that in urban areas for primary school children, and, in middle school, much fewer rural students performed in the “excellent” rank, and more rural girls failed in the cardiorespiratory fitness overall assessment (*p* < 0.01).

Muscular Fitness: Muscular fitness represents one’s muscle strength and endurance for large muscle groups. Generally, the results of muscular fitness showed that urban students showed greater performance in the overall muscular fitness among elementary school and middle school girls, but performed much more poorly among middle school boys when compared with rural ones (*p* < 0.01, Table 8). Concretely, the 1 min rope skip was tested among all the grades of elementary school students according to the Standard, and the results showed a much larger gap between rural and urban areas (*p* < 0.01). Students from rural towns measured an even lower scored “excellent” ranking and a relatively higher ranked “fail” proportion among all observed grades. Only students in grade 3th–4th and grade 5th–6th, as well as girls in grade 7th–9th participated in 1 min sit up test, and a larger portion of rural ones failed than those in urban areas (*p* < 0.01), where much more students performed in the “excellent” rank (*p* < 0.01). For boys in middle school, the muscular strength was tested by the pull up, and, interestingly, boys from rural areas performed much better than urban ones, with a higher rate scoring in the “excellent” rank and less in the “fail” rank(*p* < 0.01). Nevertheless, the long jump was tested in students during middle school and indicated low limb muscular explosiveness. Urban girls performed relatively better than rural girls (*p* < 0.01), while there was a slightly greater performance for rural boys (*p* < 0.01).

Flexibility: The rural–urban differences in flexibility for children and adolescents are shown in Table 9, and the sit reach test was applied to assess student flexibility. Generally, a larger amount of urban boys analyzed ranked in the “fail” level and a smaller amount ranked in the “excellent” level than rural ones (*p* < 0.01), except for grade 1st–2nd, while urban girls tested with higher percentages in the “excellent” rank and lower percentages in the “fail” rank when compared to rural girls (*p* < 0.01), except for grade 5th–6th.

### 3.2. Overweight Prevalence of Children and Adolescents and Their Urban–Rural Disparity in Physical Fitness

**Figure 2:**
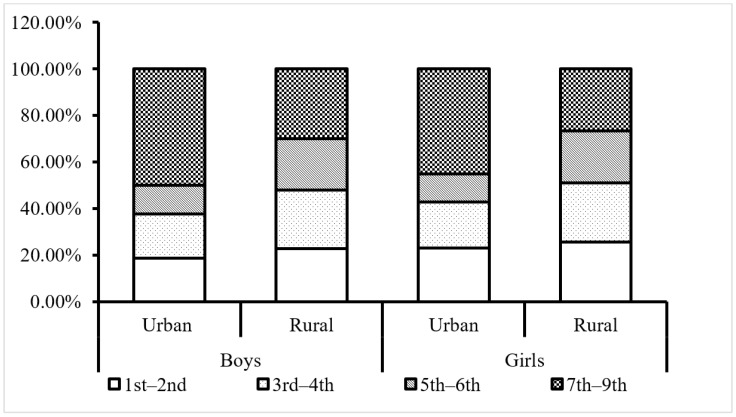
Graphic bar chart of overweight students’ distributions by grade, sex, and area of residence.

Student urban–rural Disparity in the View of Overweight Prevalence. As shown in Figure 2, a total of 35,040 children and adolescents (boys 19,555, girls 15,485) aged 7–15 years were overweight or obese from central south China, and they formed the sub-group (short for overweight below) for the overweight prevalence study. According to the crowd distribution, all the rural students constituted a larger share in the overweight population than urban students among grades 1st–2nd, 3rd–4th, and 5th–6th (*p* < 0.01), and the gaps between the urban–rural overweight distribution differences were enlarged with each grade increase. However, the overweight boys and girls in middle school were largely from urban areas, with proportions close to 50% versus no more than 30% from rural areas (χ^2^ = 811.41, 572.58 in boys and girls respectively, *p <* 0.01).

Body Composition: As summarized in Table 10, the height, body weight, and BMI of overweight students were shown and grouped by sex and resident area. All the basic data were in accordance with the law regarding students’ growth and development; meanwhile, all the boys were much taller and heavier than even-aged girls (*p* < 0.05). Generally, urban students were much taller and heavier than their rural peers among all school ages (*p* < 0.05). The criteria of “overweight or not” varies in different grades according to the Standards, and the changes in student BMI were basically consistent with height and body weight. When compared to urban students, the BMI values of rural primary school students were higher (*p* < 0.01), while the situation in urban middle schools was reversed, with fatter boys (*p* < 0.01) and no differences among girls.

Speed Agility: As is shown in Table 11, urban students in grade 1st–2nd and girls in grade 3th–4th performed better than their rural peers (*p* < 0.05) in speed agility, while the situation in middle school was reversed (*p* < 0.01). Additionally, although girls ran slower than boys in the 50 m sprint, girls had better speed agility performance (*p* < 0.01).

Cardiorespiratory Fitness: As is shown in Table 12, an urban–rural difference was detected in the overall scores and variables of cardiorespiratory fitness. Generally, the overall cardiorespiratory fitness in rural students was poorer than their urban peers (*p* < 0.01), while all boys gained much lower scores than girls in both areas (*p* < 0.01). Concretely, the vital capacity of students from urban areas was more outstanding than their rural peers (*p* < 0.05), and all boys from these two areas behaved better than girls (*p* < 0.05) except for rural grade 3rd–4th. Urban students did better in the 1 min rope skip test than rural students, while girls did better than boys (*p* < 0.05). There was no difference between the urban–rural students when testing the shuttle run, and both the boys and girls from rural areas ran faster than urban even-age ones in the middle distance race. (*p* < 0.05).

Muscular Fitness: As is shown in Table 13, an urban–rural difference was detected in the overall scores and variables of muscular fitness. Generally, the overall muscular fitness in urban area peers was better than rural peers in primary school (*p* < 0.01), while the situation was reversed in middle school boys (*p* < 0.01) and there were no differences among girls. Except for girls from rural areas performing better than boys in grade 5th–6th, the sex differences in overall muscular fitness in primary school students were not significantly different; however, middle school girls assessed better than their peer boys (*p* < 0.01). Concretely, urban students performed many more sit ups in 1 min than rural ones (*p* < 0.05), while the situation of the boys pull up test was the opposite in middle school (*p* < 0.01). The long jump was tested only in grade 7th–9th, and rural students jumped farther than urban ones (*p* < 0.01), with boys performing better than girls (*p* < 0.01). The result of the 1 min rope skip test was the same as it was in cardiorespiratory fitness.

Flexibility: As is shown in Table 14, significant rural–urban differences were observed in all grades (*p* < 0.05). Generally, girls did better than boys (*p* < 0.01), and rural boys had superior flexibility to urban ones (*p* < 0.01), while the result in girls showed the opposite trend (*p* < 0.05).

### 3.3. Physical Fitness Correlation Analysis of Overweight Prevalence

Table 15 shows the correlation between BMI, sex, urban–rural residence, and physical fitness in overweight children and adolescents. A negative association was found between BMI and physical fitness indices in all grades, except for flexibility (*p* < 0.05). The association between urban–rural residence and cardiorespiratory fitness was negative in all grades, and the *r* values were moderate in grade 1st–2nd and grade 3rd–4th (r _grade 1st–2nd_ = −0.398, r _grade 3rd–4th_ = −0.395; *p* < 0.01). The correlation between rural–urban residence and muscular fitness was negative in primary school, and the *r* values were moderate (r _grade 1st–2nd_ = −0.416, r _grade 3rd–4th_ = −0.419; r _grade 5th–6th_ = −0.335; *p* < 0.01), while it was positive in middle school, and the association between rural–urban residence and speed–agility was the same as the muscular fitness, but the *r* values were very low. The Pearson *r* values indicated that sex was negatively correlated with muscular fitness in grade 3rd–4th (*p <* 0.01) and with flexibility in primary school, while it was positively correlated with these two variants in grades 1st–2nd, 5th–6th, and 7th–9th, especially the muscular fitness in grade 7th–9th, with a moderate *r* value (r _grade 7th–9th_ *=* 0.567; *p <* 0.01). Additionally, the variant sex was positively correlated to speed agility and cardiorespiratory fitness in all grades (*p <* 0.01).

## 4. Discussion

The results of this study showed a lower overweight proportion and lower physical fitness in rural children and adolescents as compared with their urban peers, except for flexibility, which was better in rural boys than urban boys, but not better among girls. The higher flexibility in urban girls could be attributed to a greater participation in club sports, such as gymnastics or dancing, which are limited in rural settings, and the reason for higher flexibility in rural boys could be due to the lower muscular strength. The results of this study further show that urban–rural differences in physical fitness remained among overweight students, and a higher overweight epidemic level was observed in rural areas. In addition, rural–urban residence, sex, and BMI were the relevant factors for the physical fitness of overweight children and adolescents.

Studies on the differences between urban and rural areas regarding children’s and adolescents’ physical fitness have been widely discussed in many countries around the world, but the results are not the same. There are many contradictory results in the literature indicating that poorer physical fitness is more prevalent in adolescents in rural areas, as is presented as follows. In accordance with our results, measurements in Ecuador [12] and Mexico [8] found that children and adolescents in urban areas had better performance in cardiorespiratory fitness and muscular fitness than their rural counterparts. Nonetheless, contrary results have been found in Austria [9], Spain [7], and Turkey [21], where urban living environment was associated with higher body weight and lower physical fitness. Additionally, other results showed that no differences were detected in the standing long jump, counter movement jump, cardiorespiratory fitness, or sit reach test between rural and urban areas in Serbia [11]. As have been shown in other studies, these equivocal results could be partly influenced by the human development indexes [22]; for instance, there may be cultural differences among the studies, variations in the definition of urban and rural cultural environments, as well as differences for evaluating physical abilities in different countries [23]. Besides, the socioeconomic status in different counties was another one of the determinants. The physical fitness of children and adolescents in rural areas was worse than that in urban areas in central south China. The geographical location differences may be caused by the following potential factors: (1) there were much more abundant sport infrastructures and more social-friendly sports atmospheres in urban areas, which facilitate much easier accesses for youths to participate in organized sports activities than for rural youths; (2) there were much more allocated specialized physical education faculties and sports community instructors in urban areas than in rural areas.

Regarding the overweight prevalence of children and adolescents from central south China, firstly, a higher overweight epidemic level was observed in rural areas among primary school, but the overweight epidemic was more prevalent in urban middle school (grade 7th–9th). The situation in primary school reflected that the rural implementation of child nutrition improvement projects had been efficient, and there were more kids who were even met beyond their nutrition needs. As is shown in the research report on food nutrition status and countermeasures for Chinese children and adolescents, children and adolescents from rural areas had higher carbohydrate energy ratios than their urban peers, whereas they had the same level of fat energy ratios between urban peers [24]. The reason for the severe overweight phenomenon in urban middle schools might due to the intense academic burden and pressure, in addition to excess nutrition and the natural processes of growth and maturation [25]. Secondly, the muscular fitness of rural children was worse than those in the same urban grades, while grade 7th–9th showed the opposite trend. Interestingly, students from rural towns measured a higher BMI mean value in grades 1st–2nd, 3rd–4th, and 5th–6th, while urban boys in grade 7th–9th showed a higher BMI index than the rural boys, with no differences between girls. From these results, we can perhaps draw a conclusion that muscular fitness levels can be influenced by BMI and, in fact, there are available studies supporting this hypothesis [26,27,28]. Besides, it should be noted that the results of the timed rope skip and sit up were superior in urban students, whereas, in grade 7th–9th, the tested parameters were replaced by the long jump and pull up, which were performed better by rural students. The changes in the parameter setting during different growth stages could also partially explain the phenomena mentioned above. Certainly, the tested differences in body composition and physical fitness between boys and girls for overweight children and adolescents were consistent with the previous studies confirming that the overweight epidemic was more prevalent in boys [29,30,31,32,33,34], which at least partially could be attributed to the Chinese traditional social belief that, from early childhood, boys should be heavy-built and girls should be slender. In that view, parents tend to be more tolerant of excess weight and obesity in boys, and pay more attention to body shape management in girls. Moreover, the media environment, where thinness is taken as a symbol of beauty, can also explain the higher BMI values in boys when compared to girls, who, suffering from this effect and after reaching puberty, have to cater to the social aesthetic with higher requirements and standards for body shapes. Hence, the distinct results of body shape management in boys and girls lead to the clear sex differences in their cardiorespiratory fitness and muscular fitness assessments.

The results of this study further highlight the correlation factors for physical fitness of overweight children and adolescents from central south China. Excluding flexibility, speed agility, muscular fitness, and cardiorespiratory fitness were associated with rural–urban residence, sex, and BMI. As the association between BMI and physical fitness was negative, it is required to encourage students to attend sufficient amounts and intensities of prolonged engagement physical activities [10,31,32], as well as switch dietary habits [28,35,36] to improve physical fitness among overweight children and adolescents. The rural–urban residence was a moderate correlated factor of muscular fitness in grade 1st–6th students, and sex was a moderate correlation factor of muscular fitness in grade 7th–9th students. Accordingly, when building state strategies and interventions to promote physical activity and health in children and adolescents, the government and related functional departments should take the factors of rural–urban residence and the sex of students into consideration among different school stages.

## 5. Limitations

The limitations of our study include reliance on self-reported data and a lack of field investigation. Findings were based on the Students’ Physical Fitness Monitoring Data Management Center, and the original raw data of physical fitness were measured and uploaded by schools in different areas. Although we included all the valid figures, the students’ physical fitness evaluation criterions may be slightly different. Additionally, existing data were tested according to Fitness and Health Standard of Students, whose measured parameters and grading standards vary according to different grades and sex. Furthermore, the participants were recruited from a specific area of China, and physical fitness was assessed using the Chinese National Student Physical Fitness Standard, so the implication of the findings should be re-considered if they are to be generalized to children and adolescents from other regions, countries, or of different ethnicities.

## 6. Conclusions

Overall, the findings of this present study indicate that: (1) urban children and adolescents in central south China had an overall healthier profile than their rural peers, particularly in overweight groups; (2) society has witnessed the importance attached to health in urban areas, while the rural students’ physical health conditions need to be improved urgently; 3) children and adolescents in rural areas should be given special attention when building a state strategy and interventions to promote physical activity and health. Future works are needed to explore how to maintain and improve the physical fitness of children and adolescents who will coexist with COVID-19 in the long run.

## Figures and Tables

**Table 1:** Number and distribution of subjects by grade, sex, and area of residence.

Grade	Total (N)	Boys		Girls	
		Urban (N, %)	Rural (N, %)	Urban (N, %)	Rural (N, %)
1st–2nd	51,148	12,333 (46.19)	14,368 (53.81)	10,832 (44.31)	13,615 (55.69)
3rd–4th	52,368	10,884 (39.98)	16,366 (60.02)	9483 (37.75)	15,635 (62.25)
5th–6th	42,578	7298 (33.07)	14,770 (66.93)	6213 (30.29)	14,297 (69.71)
7th–9th	70,624	20,256 (54.89)	16,648 (45.11)	17,664 (52.38)	16,056 (47.62)
Total	216,718	50,771 (44.96)	62,152 (55.04)	44,192 (42.58)	59,603 (57.42)

**Table 2:** Indicator weight of overall physical fitness by grade.

Grade	BMI	Vital Capacity	50 m Run	Sit Reach	1 min Rope Skip	1 min Sit Up	Pull Up	Shuttle Run	Long Jump	800 m Run	1000 m Run
1st–2nd	15%	15%	20%	30%	20%	/	/	/	/	/	/
3rd–4th				20%	20%	10%	/	/	/	/	/
5th–6th				10%	10%	20%	/	10%	/	/	/
7th–9th				10%	/	10% (girls)	10% (boys)	/	10%	20% (girls)	20% (boys)

**Table 3:** BMI cut-points of weight status by sex and grade.

Grade	Low Weight		Normal Weight		Overweight		Obesity	
	Boys	Girls	Boys	Girls	Boys	Girls	Boys	Girls
1st	≤13.4	≤13.2	13.5~18.1	13.3~17.3	18.2~20.3	17.4~19.2	≥20.4	≥19.3
2nd	≤13.6	≤13.4	13.7~18.4	13.5~17.8	18.5~20.4	17.9~20.2	≥20.5	≥20.3
3rd	≤13.8	≤13.5	13.9~19.4	13.6~18.6	19.5~22.1	18.7~21.1	≥22.2	≥21.2
4th	≤14.1	≤13.6	14.2~20.1	13.7~19.4	20.2~22.6	19.5~22.0	≥22.7	≥22.1
5th	≤14.3	≤13.7	14.4~21.4	13.8~20.5	21.5~24.1	20.6~22.9	≥24.2	≥23.0
6th	≤14.6	≤14.1	14.7~21.8	14.2~20.8	21.9~24.5	20.9~23.6	≥24.6	≥23.7
7th	≤15.4	≤14.7	15.5~22.1	14.8~21.7	22.2~24.9	21.8~24.4	≥25.0	≥24.5
8th	≤15.6	≤15.2	15.7~22.5	15.3~22.2	22.6~25.2	22.3~24.8	≥25.3	≥24.9
9th	≤15.7	≤15.9	15.8~22.8	16.0~22.6	22.9~26.0	22.7~25.1	≥26.1	≥25.2

**Table 4:** Number and ranked distribution of overall fitness in participants by grade, sex, and area of residence.

Grade	Sex	Excellent		Good		Pass		Fail	
		Urban (N, %)	Rural (N, %)	Urban (N, %)	Rural (N, %)	Urban (N, %)	Rural (N, %)	Urban (N, %)	Rural (N, %)
1st–2nd	Boys	2545 (20.64)	465 (3.24) **	3366 (27.29)	3013 (20.97)	6045 (49.01)	9768 (67.98)	377 (3.06)	1122 (7.81) **
	Girls	2610 (24.10)	505 (3.71) **	4415 (40.76)	3723 (27.34)	3676 (33.94)	8649 (63.53)	131 (1.21)	738 (5.42) **
	Total	5155 (22.26)	970 (3.47) **	7781 (33.59)	6736 (24.07)	9721 (41.96)	18,417 (65.81)	508 (2.19)	1860 (6.65) **
3rd–4th	Boys	2716 (24.95)	685 (4.19) **	3169 (29.12)	3209 (19.61)	4769 (43.82)	11,251 (68.75)	230 (2.11)	1221 (7.46) **
	Girls	2045 (21.56)	487 (3.11) **	3723 (39.26)	3727 (23.84)	3619 (38.16)	10,361 (66.27)	96 (1.01)	1060 (6.78) **
	Total	4761 (23.38)	1172 (3.66) **	6892 (33.84)	6936 (21.67)	8388 (41.18)	21,582 (67.44)	326 (1.60)	2281 (7.13) **
5th–6th	Boys	1337 (18.32)	454 (3.07) **	2298 (31.49)	3314 (22.44)	3486 (47.77)	9960 (67.43)	177 (2.43)	1042 (7.05) **
	Girls	1006 (16.19)	440 (3.08) **	2651 (42.67)	4095 (28.64)	2466 (39.69)	9060 (63.37)	90 (1.45)	702 (4.91) **
	Total	2343 (17.34)	894 (3.08) **	4949 (36.63)	7409 (25.49)	5952 (44.05)	19,020 (65.44)	267 (1.98)	1744 (6.00) **
7th–9th	Boys	420 (2.07)	422 (2.53) *	2841 (14.03)	2794 (16.78)	13,133 (64.84)	11,400 (68.48)	3862 (19.07)	2032 (12.21) **
	Girls	882 (4.99)	608 (3.79) **	6363 (36.02)	5080 (31.64)	9612 (54.42)	9551 (59.49)	807 (4.57)	817 (5.09) *
	Total	1302 (3.43)	1030 (3.15) **	9204 (24.27)	7874 (24.08)	22,745 (59.98)	20,978 (64.15)	4669 (12.31)	2849 (8.72) *
Total	Boys	7018 (13.82)	2026 (3.26) **	11,674 (22.99)	12,330 (19.84)	27,433 (54.03)	42,379 (68.18)	4646 (9.15)	5417 (8.72) *
	Girls	6543 (14.81)	2040 (3.42) **	17,152 (38.81)	16,625 (27.89)	19,373 (43.84)	37,621(63.12)	1124 (2.54)	3317 (5.57) **
	Total	13,561 (14.28)	4066 (3.34) **	28,826 (30.35)	28,955 (23.78)	46,806 (49.29)	80,000 (65.71)	5770 (6.08)	8734 (7.17) **

* *p* < 0.05, ** *p* < 0.01; when comparing the difference in proportions of each ranked distribution between urban and rural students, only the proportions of “fail” and “excellent” rankings were evaluated here by the chi-square test.

**Table 5:** Number and ranked distribution of body composition in participants by grade, sex, and area of residence.

Grade	Sex	Low Weight		Normal Weight		Overweight		Obesity	
		Urban (N, %)	Rural (N, %)	Urban (N, %)	Rural (N, %)	Urban (N, %)	Rural (N, %)	Urban (N, %)	Rural (N, %)
1st–2nd	Boys	1006 (8.16)	1581 (11.00) **	9329 (75.64)	10,764 (74.91)	1137 (9.22)	1013 (7.05) **	861 (6.98)	1010 (7.03)
	Girls	1119 (10.33)	1498 (11.00)	8163 (75.36)	9885 (72.60)	1021 (9.43)	1320 (9.70)	529 (4.88)	912 (6.70) **
3rd–4th	Boys	1010 (9.28)	2147 (13.12) **	7859 (72.21)	11,984 (73.22)	1298 (11.93)	1250 (7.64) **	717 (6.59)	985 (6.02)
	Girls	913 (9.63)	1711 (10.94) *	7239 (76.34)	11,702 (74.84)	906 (9.55)	1317 (8.42) *	425 (4.48)	905 (5.79) **
5th–6th	Boys	580 (7.95)	1691 (11.45) **	5391 (73.87)	11,110 (75.22)	874 (11.98)	1102 (7.46) **	453 (6.21)	867 (5.87)
	Girls	397 (6.39)	1078 (7.54) *	4989 (80.30)	11,253 (78.71)	557 (8.97)	1117 (7.81) *	270 (4.35)	849 (5.94) **
7th–9th	Boys	1398 (6.90)	1674 (10.06) **	13,521 (66.75)	12,326 (74.04)	2929 (14.46)	1586 (9.53) **	2408 (11.89)	1062 (6.38) **
	Girls	914 (5.17)	1017 (6.33) **	13,715 (77.64)	12,717 (79.20)	1845 (10.44)	1374 (8.56) **	1190 (6.74)	948 (5.90) *

* *p* < 0.05, ** *p* < 0.01; when comparing the difference in proportions of each ranked distribution between urban and rural students, only the proportions of “low weight”, “overweight”, and “obesity” rankings were evaluated here by the chi-square test.

**Table 6:** Number and ranked distribution of speed agility in participants grouped by grade, sex, and area of residence.

Indicators	Grade	Sex	Excellent		Good		Pass		Fail	
			Urban (N, %)	Rural (N, %)	Urban (N, %)	Rural (N, %)	Urban (N, %)	Rural (N, %)	Urban (N, %)	Rural (N, %)
Sprint	1st–2nd	Boys	2241 (18.17)	2249 (15.65) **	812 (6.58)	931 (6.48)	8146 (66.05)	8931 (62.16)	1134 (9.19)	2257 (15.71) **
		Girls	2985 (27.56)	3870 (28.42)	2379 (21.96)	2545 (18.69)	5103 (47.11)	6254 (45.93)	365 (3.37)	946 (6.95) **
	3rd–4th	Boys	1882 (17.29)	2329 (14.23) **	884 (8.12)	1085 (6.63)	7326 (67.31)	10,716 (65.48)	792 (7.28)	2236 (13.66) **
		Girls	977 (10.30)	1907 (12.20) **	2028 (21.39)	2917 (18.66)	6072 (64.03)	9460 (60.51)	406 (4.28)	1351 (8.64) **
	5th–6th	Boys	1338 (18.33)	2410 (16.32) **	534 (7.32)	990 (6.70)	4827 (66.14)	9852 (66.70)	599 (8.21)	1518 (10.28) **
		Girls	504 (8.11)	1713 (11.98) **	1250 (20.12)	2920 (20.42)	4075 (65.59)	8668 (60.63)	384 (6.18)	996 (6.97) *
	7th–9th	Boys	5202 (25.68)	3928 (23.59) **	1758 (8.68)	1384 (8.31)	11,777 (58.14)	10,242 (61.52)	1519 (7.50)	1094 (6.57) **
		Girls	2149 (12.17)	3152 (19.63) **	3881 (21.97)	3939 (24.53)	10,666 (60.38)	8315 (51.79)	968 (5.48)	650 (4.05) **

* *p* < 0.05, ** *p* < 0.01; when comparing the difference in proportions of each ranked distribution between urban and rural students, only the proportions of “excellent” and “fail” rankings were evaluated here by the chi-square test.

**Table 7:** Number and ranked distribution of cardiorespiratory fitness indicators in participants grouped by grade, sex, and area of residence.

Indicators	Grade	Sex	Excellent		Good		Pass		Fail	
			Urban (N, %)	Rural (N, %)	Urban (N, %)	Rural (N, %)	Urban (N, %)	Rural (N, %)	Urban (N, %)	Rural (N, %)
Vital capacity	1st–2nd	Boys	1825 (14.80)	1126 (7.84) **	2632 (21.34)	1631 (11.35)	7567 (61.36)	10,073 (70.11)	309 (2.51)	1538 (10.70) **
		Girls	3856 (35.60)	3172 (23.30) **	2931 (27.06)	3111 (22.85)	3906 (36.06)	6413 (47.10)	139 (1.28)	919 (6.75) **
	3rd–4th	Boys	1246 (11.45)	897 (5.48) **	2952 (27.12)	2710 (16.56)	6289 (57.78)	11,016 (67.31)	398 (3.66)	1740 (10.63) **
		Girls	3323 (35.04)	3801 (24.31) **	1904 (20.08)	2731 (17.47)	4135 (43.60)	8197 (52.43)	121 (1.28)	906 (5.79) **
	5th–6th	Boys	807 (11.06)	1046 (7.08) **	2090 (28.64)	1981 (13.41)	4111 (56.33)	9572 (64.81)	290 (3.97)	2171 (14.70) **
		Girls	2607 (41.96)	3354 (23.46) **	1130 (18.19)	2246 (15.71)	2403 (38.68)	7765 (54.31)	73 (1.17)	932 (6.52) **
	7th–9th	Boys	4701 (23.21)	1209 (7.26) **	3798 (18.75)	2101 (12.62)	10,123 (49.98)	9943 (59.72)	1634 (8.07)	3395 (20.39) **
		Girls	6916 (39.15)	3948 (24.59) **	2795 (15.82)	2017 (12.56)	7320 (41.44)	8134 (50.66)	633 (3.58)	1957 (12.19) **
1 min rope skip	1st–2nd	Boys	4615 (37.42)	1115 (7.76) **	1453 (11.78)	1251 (8.71)	6105 (49.50)	10,421 (72.53)	160 (1.30)	1581 (11.00) **
		Girls	3741 (34.54)	774 (5.68) **	1927 (17.79)	1553 (11.41)	5075 (46.85)	9953 (73.10)	89 (0.82)	1335 (9.81) **
	3rd–4th	Boys	5180 (47.59)	1710 (10.45) **	1338 (12.29)	1327 (8.11)	4260 (39.14)	11,694 (71.45)	106 (0.97)	1635 (9.99) **
		Girls	4085 (43.08)	1206 (7.71) **	1784 (18.81)	1587 (10.15)	3563 (37.57)	11,311 (72.34)	51 (0.54)	1531 (9.79) **
	5th–6th	Boys	2804 (38.42)	1169 (7.91) **	1082 (14.83)	1082 (7.33)	3237 (44.35)	10,758 (72.84)	175 (2.40)	1761 (11.92) **
		Girls	2199 (35.39)	962 (6.73) **	1313 (21.13)	1303 (9.11)	2627 (42.28)	10,600 (74.14)	74 (1.19)	1432 (10.02) **
Shuttle run	5th–6th	Boys	1089 (14.92)	2105 (14.25) *	1061 (14.54)	1835 (12.42)	4603 (63.07)	9096 (61.58)	545 (7.47)	1734 (11.74) **
		Girls	1180 (18.99)	3239 (22.66) **	1055 (16.98)	2344 (16.40)	3752 (60.39)	7568 (52.93)	226 (3.64)	1146 (8.02) **
Middle distance race	7th–9th	Boys	2161 (10.67)	2827 (16.98) **	2708 (13.37)	2550 (15.32)	10,058 (49.65)	8394 (50.42)	5329 (26.31)	2877 (17.28) **
		Girls	2335 (13.22)	2421 (15.08) **	3217 (18.21)	2974 (18.52)	9686 (54.83)	8435 (52.53)	2426 (13.73)	2226 (13.86)
Cardiorespiratory fitness	1st–2nd	Boys	2612 (21.18)	380 (2.64) **	3086 (25.04)	1354 (9.42)	6354 (51.52)	10,337 (71.94)	281 (2.28)	2297 (15.99) **
		Girls	2837 (26.19)	504 (3.70) **	3324 (30.69)	2001 (14.70)	4531 (41.83)	9378 (68.88)	140 (1.29)	1732 (12.72) **
	3rd–4th	Boys	2814 (25.85)	720 (4.40) **	3314 (30.45)	1736 (10.61)	4543 (41.74)	11,373 (69.49)	213 (1.96)	2537 (15.50) **
		Girls	2914 (30.73)	841 (5.38) **	3169 (33.42)	2510 (16.05)	3278 (34.57)	10,216 (65.34)	122 (1.29)	2068 (13.23) **
	5th–6th	Boys	389 (5.33)	557 (3.77) **	1576 (21.59)	2219 (15.02)	4779 (65.48)	9170 (62.09)	554 (7.59)	2824 (19.12) **
		Girls	852 (13.71)	1723 (12.05) **	2244 (36.12)	3608 (25.24)	2904 (46.74)	7398 (51.75)	213 (3.43)	1568 (10.97) **
	7th–9th	Boys	1240 (6.12)	805 (4.84) **	3436 (16.96)	2583 (15.52)	10,688 (52.76)	9189 (55.20)	4892 (24.15)	4071 (24.45)
		Girls	1805 (10.22)	1358 (8.46) **	4755 (26.92)	3439 (21.42)	8931 (50.56)	8223 (51.21)	2173 (12.30)	3036 (18.91) **

* *p* < 0.05, ** *p* < 0.01; when comparing the difference in proportions of each ranked distribution between urban and rural students, only the proportions of “excellent” and “fail” rankings were evaluated here by the chi-square test.

**Table 8:** Number and ranked distribution of muscular fitness indicators in participants grouped by grade, sex, and area of residence.

Indicators	Grade	Sex	Excellent		Good		Pass		Fail	
			Urban (N, %)	Rural (N, %)	Urban (N, %)	Rural (N, %)	Urban (N, %)	Rural (N, %)	Urban (N, %)	Rural (N, %)
1 min rope-skip	1st–2nd	Boys	4615 (37.42)	1115 (7.76) **	1453 (11.78)	1251 (8.71)	6105 (49.50)	10,421 (72.53)	160 (1.30)	1581 (11.00) **
		Girls	3741 (34.54)	774 (5.68) **	1927 (17.79)	1553 (11.41)	5075 (46.85)	9953 (73.10)	89 (0.82)	1335 (9.81) **
	3rd–4th	Boys	5180 (47.59)	1710 (10.45) **	1338 (12.29)	1327 (8.11)	4260 (39.14)	11,694 (71.45)	106 (0.97)	1635 (9.99) **
		Girls	4085 (43.08)	1206 (7.71) **	1784 (18.81)	1587 (10.15)	3563 (37.57)	11,311 (72.34)	51 (0.54)	1531 (9.79) **
	5th–6th	Boys	2804 (38.42)	1169 (7.91) **	1082 (14.83)	1082 (7.33)	3237 (44.35)	10,758 (72.84)	175 (2.40)	1761 (11.92) **
		Girls	2199 (35.39)	962 (6.73) **	1313 (21.13)	1303 (9.11)	2627 (42.28)	10,600 (74.14)	74 (1.19)	1432 (10.02) **
Pull-up/1 min Sit-up	3rd–4th	Boys	1304 (11.98)	1313 (8.02) **	1836 (16.87)	2103 (12.85)	7357 (67.59)	11,736 (71.71)	387 (3.56)	1214 (7.42) **
		Girls	1109 (11.69)	1133 (7.25) **	1662 (17.53)	1996 (12.77)	6380 (67.28)	11,223 (71.78)	332 (3.50)	1283 (8.21) **
	5th–6th	Boys	1436 (19.68)	1752 (11.86) **	1457 (19.96)	2370 (16.05)	4168 (57.11)	9734 (65.90)	237 (3.25)	914 (6.19) **
		Girls	1081 (17.40)	1558 (10.90) **	1301 (20.94)	2045 (14.30)	3740 (60.20)	9835 (68.79)	91 (1.46)	859 (6.01) **
	7th–9th	Boys	387 (1.91)	766 (4.60) **	317 (1.56)	720 (4.32)	3580 (17.67)	5806 (34.88)	15,972 (78.85)	9356 (56.20) **
		Girls	1775 (10.05)	776 (4.83) **	3382 (19.15)	2086 (12.99)	12,053 (68.23)	11,805 (73.52)	454 (2.57)	1389 (8.65) **
Long-jump	7th–9th	Boys	1375 (6.79)	1072 (6.44)	2568(12.68)	2283 (13.71)	12,190(60.18)	9652 (57.98)	4123 (20.35)	3641 (21.87) **
		Girls	2160 (12.23)	3063 (19.08) **	3456 (19.57)	3843 (23.93)	9794 (55.45)	7782 (48.47)	2254 (12.76)	1368 (8.52) **
Muscular fitness	1st–2nd	Boys	/	/	/	/	/	/	/	/
		Girls	/	/	/	/	/	/	/	/
	3rd–4th	Boys	3459 (31.78)	895 (5.47) **	2570 (23.61)	1764 (10.78)	4621 (42.46)	11,560 (70.63)	234 (2.15)	2147 (13.12) **
		Girls	2445 (25.78)	513 (3.28) **	2697 (28.44)	1743 (11.15)	4176 (44.04)	11,282 (72.16)	165 (1.74)	2097 (13.41) **
	5th–6th	Boys	1262 (17.29)	605 (4.10) **	2241 (30.71)	2250 (15.23)	3535 (48.44)	9999 (67.70)	260 (3.56)	1916 (12.97) **
		Girls	961 (15.47)	516 (3.61) **	1877 (30.21)	1872 (13.09)	3266 (52.57)	10,211 (71.42)	109 (1.75)	1698 (11.88) **
	7th–9th	Boys	133 (0.66)	239 (1.44) **	2340 (11.55)	3583 (21.52)	2736 (13.51)	3649 (21.92)	15,047 (74.28)	9177 (55.12) **
		Girls	932 (5.28)	670 (4.17) **	11,291 (63.92)	10,379 (64.64)	3587 (20.31)	3059 (19.05)	1854 (10.50)	1948 (12.13) **

** *p* < 0.01; when comparing the difference in proportions of each ranked distribution between urban and rural; only the proportions of “excellent” and “fail” rankings were evaluated here by the chi-square test.

**Table 9:** Number and ranked distribution of flexibility in participants grouped by grade, sex, and area of residence.

Indicators	Grade	Sex	Excellent		Good		Pass		Fail	
			Urban (N, %)	Rural (N, %)	Urban (N, %)	Rural (N, %)	Urban (N, %)	Rural (N, %)	Urban (N, %)	Rural (N, %)
Sit-reach	1st–2nd	Boys	2044 (16.57)	2421 (16.85)	1610 (13.05)	2228 (15.51)	8228 (66.72)	9440 (65.70)	451 (3.66)	279 (1.94) **
		Girls	2570 (23.73)	1199 (8.81) **	2043 (18.86)	1694 (12.44)	5782 (53.38)	9805 (72.02)	437 (4.03)	917 (6.74) **
	3rd–4th	Boys	1402 (12.88)	2925(17.87) **	1931 (17.74)	3848 (23.51)	7232 (66.45)	9258 (56.57)	319(2.93)	334 (2.04) **
		Girls	2013 (21.23)	1612 (10.31) **	1859 (19.60)	2444 (15.63)	5126 (54.05)	10,581 (67.68)	485 (5.11)	998 (6.38) **
	5th–6th	Boys	908 (12.44)	2799 (18.95) **	1576 (21.59)	3999 (27.08)	4523 (61.98)	7766 (52.58)	291 (3.99)	206 (1.39) **
		Girls	1299 (20.91)	1716 (12.00) **	1524 (24.53)	3059 (21.40)	3043 (48.98)	8718 (60.98)	347 (5.59)	804 (5.62)
	7th–9th	Boys	2051 (10.13)	1896 (11.39) **	2641 (13.04)	2687 (16.14)	13,319 (65.75)	11,252 (67.59)	2245 (11.08)	813 (4.88) **
		Girls	3238 (18.33)	1460 (9.09) **	2827 (16.00)	2040 (12.71)	10,032 (56.79)	11,013 (68.59)	1567 (8.87)	1543 (9.61) *

* *p* < 0.05, ** *p* < 0.01; when comparing the difference in proportions of each ranked distribution between urban and rural students, only the proportions of “excellent” and “fail” rankings were evaluated here by the chi-square test.

**Table 10:** Body composition in overweight students grouped by grade, sex, and area of residence.

Grade	Residence	Boys			Girls		
		Height (cm)	Weight (kg)	BMI (kg/m^2^)	Height (cm)	Weight (kg)	BMI (kg/m^2^)
1st–2nd	Urban	126.97 ± 0.16	33.88 ± 0.14	20.97 ± 0.08	124.22 ± 0.19 ##	30.92 ± 0.20 ##	19.98 ± 0.13 ##
	Rural	122.52 ± 0.19 **	32.27 ± 0.16 **	21.41 ± 0.09 **	120.82 ± 0.18 **##	30.14 ± 0.16 *##	20.59 ± 0.1 **##
3rd–4th	Urban	138.49 ± 0.16	43 ± 0.16	22.34 ± 0.07	137.14 ± 0.21 ##	40.52d ± 0.20 ##	21.48 ± 0.08 ##
	Rural	134.82 ± 0.17 **	42.2 ± 0.19 *	23.12 ± 0.08 **	134.30 ± 0.17 **#	40.11 ± 0.18 ##	22.12 ± 0.08 **##
5th–6th	Urban	150.17 ± 0.22	54.78 ± 0.26	24.19 ± 0.09	150.15 ± 0.26	52.26 ± 0.28 ##	23.11 ± 0.09 ##
	Rural	146.57 ± 0.20 **	53.65 ± 0.23 *	24.85 ± 0.08 **	146.35 ± 0.21 **	51.77 ± 0.26 ##	24.03 ± 0.09 **##
7th–9th	Urban	164.98 ± 0.12	70.67 ± 0.16	25.85 ± 0.43	158.91 ± 0.11 ##	63.29 ± 0.17 ##	25 ± 0.05 ##
	Rural	159.58 ± 0.20 **	65.47 ± 0.22 **	25.58 ± 0.56 **	155.32 ± 0.17 **##	60.49 ± 0.20 **##	25 ± 0.06 ##

* *p* < 0.05, ** *p* < 0.01; when comparing the difference in values of each parameter between urban and rural students, # *p* < 0.05, ## *p* < 0.01; when comparing the difference in values of each parameter between boys and girls, two-way ANOVA was applied to assess the effect of factors of gender and area of residence (the interaction effect of the two factors was not considered), and multiple comparisons were further conducted to assess differences between intra- and inter-groups.

**Table 11:** Speed agility in overweight students grouped by grade, sex, and area of residence.

Grade	Residence	Boys		Girls	
		Sprint (s)	Speed Agility	Sprint (s)	Speed Agility
1st–2nd	Urban	11.34 ± 0.03	67.53 ± 21.13	11.53 ± 0.03 ##	78.11 ± 16.78 ##
	Rural	11.50 ± 0.04 **	64.53 ± 26.74 **	11.71 ± 0.04 **##	74.33 ± 24.50 **##
3rd–4th	Urban	10.37 ± 1.07	66.74 ± 19.84	10.50 ± 0.93 ##	70.94 ± 15.00 ##
	Rural	10.42 ± 1.31	65.84 ± 22.06	10.61 ± 1.55 *##	69.63 ± 20.95 *##
5th–6th	Urban	9.79 ± 1.01	65.94 ± 19.97	9.85 ± 0.95	70.05 ± 15.97 ##
	Rural	9.77 ± 1.30	67.00 ± 21.91	9.86 ± 1.33 #	69.67 ± 19.93 ##
7th–9th	Urban	8.78 ± 1.38	70.68 ± 21.88	9.55 ± 1.39 ##	71.08 ± 18.91
	Rural	8.58 ± 1.07 **	73.72 ± 19.07 **	9.11 ± 1.11 **##	76.53 ± 17.05 **##

* *p* < 0.05, ** *p* < 0.01; when comparing the difference in values of each parameter between urban and rural students, # *p* < 0.05, ## *p* < 0.01; when comparing the difference in values of each parameter between boys and girls, two-way ANOVA was applied to assess the effect of factors of sex and area of residence (the interaction effect of the two factors was not considered), and multiple comparisons were further conducted to assess differences between intra- and inter-groups.

**Table 12:** Cardiorespiratory fitness in overweight students grouped by grade, sex, and area of residence.

Grade	Residence	Boys				Girls			
		Vital Capacity (mL)	Shuttle-Run/Middle-Distance-Race (s)	1 min Rope-Skip (N)	Cardiorespiratory Fitness	Vital Capacity (mL)	Shuttle-Run/Middle-Distance-Race (s)	1 min Rope-Skip (N)	Cardiorespiratory Fitness
1st–2nd	Urban	1378.19 ± 9.07	/	87.92 ± 0.90	79.09 ± 10.64	1265.61 ± 10.25 ##	/	91.23 ± 0.94 #	82.23 ± 10.05 ##
	Rural	1294.47 ± 20.36 **	/	55.19 ± 0.77 **	67.31 ± 13.04 **	1250.91 ± 19.72	/	57.30 ± 0.74 **#	70.88 ± 12.92 **##
3rd–4th	Urban	1824.14 ± 12.0	/	111.46 ± 0.8	80.95 ± 10.37	1665.62 ± 13.64 ##	/	117.90 ± 0.92 ##	83.46 ± 10.14 ##
	Rural	1640.82 ± 18.18 **	/	77.95 ± 0.71 **	68.07 ± 14.64 **	1661.30 ± 22.70	/	84.68 ± 0.78 **##	70.79 ± 14.99 **##
5th–6th	Urban	2405.21 ± 15.79	120.87 ± 0.44	128.48 ± 0.87	74.42 ± 10.52	2287.76 ± 20.53 ##	120.36 ± 0.53	131.03 ± 0.99	80.97 ± 10.55 ##
	Rural	2074.58 ± 18.25 **	120.03 ± 0.49	114.06 ± 0.83 **	67.78 ± 15.0 **	1966.05 ± 18.81 **##	120.77 ± 0.52	117.18 ± 0.77 **#	74.82 ± 15.09 **##
7th–9th	Urban	3404.33 ± 15.43	306.53 ± 0.74	/	69.32 ± 15.46	2766.57 ± 14.33 ##	269.47 ± 0.65	/	75.19 ± 14.02 ##
	Rural	2736.84 ± 16.19**	287.20 ± 0.91 **	/	67.93 ± 14.96 **	2369.61 ± 14.3 3**##	266.41 ± 0.83 *	/	71.31 ± 15.81 **##

* *p* < 0.05, ** *p* < 0.01; when comparing the difference in values of each parameter between urban and rural, # *p* < 0.05, ## *p* < 0.01; when comparing the difference in values of each parameter between boys and girls, two-way ANOVA was applied to assess the effect of factors of sex and area of residence (the interaction effect of the two factors was not considered), and multiple comparisons were further conducted to assess differences between intra- and inter-groups.

**Table 13:** Muscular fitness in overweight students grouped by grade, sex, and area of residence.

Grade	Residence	Boys				Girls			
		Long Jump (mL)	1 min Rope Skip (N)	Pull Up/Sit Up (N)	Muscular Fitness	Long Jump (mL)	1 min Rope Skip (N)	Pull Up/Sit Up (N)	Muscular Fitness
1st–2nd	Urban	/	87.92 ± 0.90	/	80.09 ± 15.09	/	91.23 ± 0.94 #	/	80.83 ± 12.94
	Rural	/	55.19 ± 0.77 **	/	66.56 ± 18.33 **	/	57.30 ± 0.74 **#	/	66.64 ± 17.08 **
3rd–4th	Urban	/	111.46 ± 0.8	28.87 ± 0.20	78.97 ± 12.01	/	117.90 ± 0.92 ##	29.60 ± 0.26 #	79.21 ± 11.20
	Rural	/	77.95 ± 0.71 **	27.07 ± 0.20 **	67.51 ± 16.02 **	/	84.68 ± 0.78 **##	27.31 ± 0.21 **	67.86 ± 15.70 **
5th–6th	Urban	/	128.48 ± 0.87	33.65 ± 0.26	76.39 ± 11.31	/	131.03 ± 0.99	34.10 ± 0.33	77.11 ± 10.51
	Rural	/	114.06 ± 0.83 **	30.07 ± 0.24 **	67.07 ± 15.40 **	/	117.18 ± 0.77 **#	30.05 ± 0.22 **	68.00 ± 13.54 **#
7th–9th	Urban	181.5 ± 0.36	/	1.59 ± 0.04	37.89 ± 18.62	159.24 ± 0.38 ##	/	34.29 ± 0.14 ##	69.32 ± 12.47 ##
	Rural	182.9 ± 0.49 *	/	3.69 ± 0.08 **	49.14 ± 18.38 **	166.13 ± 0.45 **##	/	31.13 ± 0.21 **##	69.36 ± 14.24 ##

* *p* < 0.05, ** *p* < 0.01; when comparing the difference in values of each parameter between urban and rural students, # *p* < 0.05, ## *p* < 0.01; when comparing the difference in values of each parameter between boys and girls, two-way ANOVA was applied to assess the effect of factors of sex and area of residence (the interaction effect of the two factors was not considered), and multiple comparisons were further conducted to assess differences between intra- and inter-groups.

**Table 14:** Flexibility in overweight students grouped by grade, sex, and area of residence.

Grade	Residence	Boys		Girls	
		Sit Reach (cm)	Flexibility	Sit Reach (cm)	Flexibility
1st–2nd	Urban	7.85 ± 0.12	73.82 ± 14.86	11.89 ± 0.13 ##	77.42 ± 14.44 ##
	Rural	8.48 ± 0.14 **	75.50 ± 12.83 **	9.5 ± 0.13 **##	71.33 ± 13.74 **##
3rd–4th	Urban	6.83 ± 0.2	74.32 ± 13.65	10.83 ± 0.16 ##	76.59 ± 15.36 ##
	Rural	8.56 ± 0.13 **	77.36 ± 12.50 **	10.55 ± 0.14 **##	72.48 ± 14.78 **##
5th–6th	Urban	6.02 ± 0.16	74.43 ± 14.31	11.37 ± 0.22 ##	75.99 ± 17.53 ##
	Rural	8.23 ± 0.15 **	78.42 ± 12.0 **	10.49 ± 0.16 *##	73.10 ± 15.58 **##
7th–9th	Urban	6.21 ± 0.10	68.30 ± 20.32	12.26 ± 0.14 ##	72.31 ± 20.17 ##
	Rural	8.07 ± 0.12 **	72.82 ± 14.81 **	10.61 ± 0.14 **##	69.07 ± 17.88 **##

* *p* < 0.05, ** *p* < 0.01; when comparing the difference in values of each parameter between urban and rural students, ## *p* < 0.01; when comparing the difference in values of each parameter between boys and girls, two-way ANOVA was applied to assess the effect of factors of sex and area of residence (the interaction effect of the two factors was not considered), and multiple comparisons were further conducted to assess differences between intra- and inter-groups.

**Table 15:** The correlation between BMI, sex, urban–rural residence, and physical fitness.

Sex	Physical Fitness	BMI	Residence	Sex
1st–2nd	Speed agility	−0.058 **	−0.084 **	0.217 **
	Muscular fitness	−0.017 **	−0.416 **	0.008 **
	Cardiorespiratory fitness	−0.054 **	−0.398 **	0.083 **
	Flexibility	−0.008 **	−0.083 **	−0.031 *
3rd–4th	Speed agility	−0.071**	−0.089 **	0.07 **
	Muscular fitness	−0.012 **	−0.419 **	−0.024 **
	Cardiorespiratory fitness	−0.071 **	−0.395 **	0.067 **
	Flexibility	0.007	0.047 **	−0.045 **
5th–6th	Speed agility	−0.078 **	−0.027 **	0.036 **
	Muscular fitness	−0.064 **	−0.335 **	0.009 **
	Cardiorespiratory fitness	−0.017 *	−0.169 **	0.210 **
	Flexibility	0.044 **	0.039 *	−0.069 **
7th–9th	Speed agility	−0.077 **	0.047 **	0.015 **
	Muscular fitness	−0.193 **	0.128 **	0.567 **
	Cardiorespiratory fitness	−0.108 **	−0.035 **	0.151 **
	Flexibility	−0.075 **	0.044 **	0.036 **

* *p* < 0.05, ** *p* < 0.01 when analyzing *r* values of the correlation between variants according to the Pearson method.

## Data Availability

Based on the consent form indicating that the data will not be shared publicly for confidentiality, we will not share our data with the public.

## Supplementary Materials

The following supporting information can be downloaded at: https://www.mdpi.com/article/10.3390/ijerph20032390/s1.
